# Potential Prognostic Protein Biomarkers in Tears From Noninfectious Uveitis Patients Under Biologic Treatment as a Prelude to Personalized Medicine

**DOI:** 10.1167/iovs.65.13.29

**Published:** 2024-11-14

**Authors:** Lorena Rodríguez-Martínez, Carmen Antía Rodríguez-Fernández, Olalla Rodríguez Lemos, Begoña de Domingo, Pere García Bru, Jesús Mateos, Anxo Fernández-Ferreiro

**Affiliations:** 1 FarmaCHUSLab Group, Health Research Institute of Santiago de Compostela (IDIS), Santiago de Compostela, Spain; 2 Department of Ophthalmology, Bellvitge University Hospital, 08907 Hospitalet de Llobregat, Spain; 3 Ophthalmology Department, University Clinical Hospital of Vigo (SERGAS), Vigo, Spain; 4 Ophthalmology Department, University Clinical Hospital of Santiago Compostela (SERGAS), Santiago de Compostela, Spain; 5 Pharmacy Department, University Clinical Hospital of Santiago de Compostela (SERGAS), Santiago de Compostela, Spain

**Keywords:** noninfectious uveitis, tear proteomics, anti-TNF, biomarkers, personalized medicine

## Abstract

**Purpose:**

Adalimumab (ADA) is a systemic biological treatment option approved for the treatment of noninfectious uveitis (NIU); however, up to 40% of patients do not respond to the drug, either in a primary or secondary manner. Here, we evaluated the proteomic profile of patients with NIU who fail to ADA to identify proteins implicated in intraocular inflammation, as well as potential biomarkers for treatment response and novel therapeutic targets.

**Methods:**

Cross-sectional observational study of patients with NIU under ADA treatment for six or more months. Tears were collected with microcapillary tubes and protein analyzed by data-independent acquisition/sequential window acquisition of all theoretical mass spectra. Differentially expressed proteins (DEPs) were defined based on the fold change between their expression in nonresponders (NR) and responders (R). Protein network and gene ontology analysis were performed. The χ^2^ test for trend and receiver operating characteristic (ROC) curves were used to evaluate potential biomarkers of treatment response.

**Results:**

Twenty-nine DEPs, 14 upregulated and 15 downregulated, were detected in NR. These proteins were mainly related to enhanced neutrophil effector functions and redox imbalance. ROC analysis identified defensin-1,3 (DEF-1,3), biotinidase, and ATP-binding cassette transporter A1 as potential biomarkers for treatment response.

**Conclusions:**

This is the first study on a clinical cohort of patients with noninfectious uveitis that identifies tear proteins related to neutrophil hyperactivation as drivers of the persistent intraocular inflammation observed in NR to ADA and provides evidence that targeting interleukin 6, Janus kinases, or the complement cascade could be potential alternative therapeutic strategies in these patients. Our results indicate the potential of high-throughput proteomics to provide insights into the underlying pathological mechanisms of persistent intraocular inflammation observed in patients who do not adequately respond to anti-TNF treatment and the value of tear proteomics as a tool for personalized medicine.

Uveitis is defined as the inflammation of the uvea that includes the choroid, the ciliary body, and the iris. Noninfectious uveitis (NIU) accounts for 70% to 90% of uveitis cases^1^ and is the third-leading cause of preventable blindness in the developed world, representing a serious health concern.^2^^,^^3^

Mainstay therapy includes corticosteroids and immunosuppressants; however, the emergence of biologic agents has significantly broadened the array of treatment options.^4^ Biologic therapy offers notable effectiveness, favorable safety profiles, and faster onset of therapeutic benefits when compared to conventional immunosuppressive drugs. Among these biologics, adalimumab (ADA), a monoclonal antibody designed to target TNF-α, stands as the sole systemic biologic treatment option approved by the Food and Drug Administration and the European Medicines Agency for the treatment of noninfectious intermediate, posterior, and panuveitis.^5^ However, up to 40% of patients do not respond to the drug, either in a primary or secondary manner.^6^ Nonresponder patients are under therapeutic gap, because effective therapeutic options after anti-TNF therapy are scarce and administered off-label, leading to recurrent disease relapses, severe visual impairment, and, eventually, blindness. A change to another biological drug with a different target is usually recommended^7^ but the choice is often arbitrary, based on internal hospital protocols, physician preference, drug availability, and economic costs, lacking solid scientific evidence and standardization.

In the last decade, “shotgun” proteomics has become a key tool in clinical research. The term “shotgun” refers to the massive identification of proteins in a sample, enabling biomarker discovery, molecular diagnosis, treatment response prediction and advancing personalized medicine.^8^ Through analyzing differentially expressed proteins (DEPs) and molecular pathways, proteomics provides insights into disease mechanisms and potential new therapeutic targets. In the early stages of biomarker discovery, the use of untargeted shotgun proteomics is preferred because they maximize protein coverage (identification), and no prior knowledge of the molecular mechanisms of the disease is needed. Prior studies in patients and healthy volunteers have detected several proteins in tear samples either with data-dependent analysis (DDA) or data-independent acquisition (DIA) of the raw proteomic data.^9^^–^^11^ This amount of information leads to a broader comprehensive picture of the normal/pathological state. Velez et al.^12^ demonstrated this in a rare disease with unknown molecular mechanisms characterized by progressive uveitis, an inflammatory vitreoretinopathy where conventional treatments such as anti-TNFα or steroids had low response rates. They detected normal TNF-α levels, explaining previous treatment failures, whereas high levels of VEGF shifted the therapeutic approach toward the use of antiangiogenic drugs, resulting in remarkable clinical improvement. Additionally, high interleukin 6 (IL-6) levels in a subset of patients guided a repositioning strategy with anti-IL-6 to target fibrosis, promoting tissue repair.

A key factor in translational approaches is noninvasive sample collection. Blood is widely used for biomarkers because of its accessibility but may not accurately reflect ophthalmic diseases. Ocular fluids, such as tears and aqueous and vitreous humor, may be more representative of undergoing biological processes.^8^^,^^13^ Tears stand out as a source of biomarkers for their noninvasiveness and easy access, providing insights into the status of ocular conditions such as NIU, dry eye disease, keratoconus, and thyroid-associated orbitopathy.^11^^,^^14^

Our main objective was to use shotgun DIA proteomics to characterize the proteomic signature driving ocular inflammation in NIU patients unresponsive to anti-TNF-α. We aimed to identify potential biomarkers for treatment response and novel therapeutic targets to enhance clinical outcomes.

## Material and Methods

### Study Design

This was a multicenter, observational, cross-sectional study with NIU patients treated with ADA in two Spanish tertiary level hospitals (University Clinical Hospital of Santiago de Compostela and University Clinical Hospital of Vigo), recruited between June and September 2022. The study protocol was approved by the Galician Ethics Regional Committee (registration code 2021/014) and was conducted under the principles of the Declaration of Helsinki, according to the Spanish Law of Biomedical Research no. 14/2007. All patients were older than two years of age and signed the informed consent voluntarily.

Inclusion criteria were patients diagnosed with NIU that needed ADA to control intraocular inflammation, and a minimum six-month ADA treatment was required. Diagnosis was based on clinical symptoms, ophthalmological examination, and commonly requested biochemical, immunological, and hematological analysis. Subcutaneous ADA treatment was initiated due to inefficacy of first-line therapy, and refractoriness to treatment was defined as persistence of inflammation despite the use of the highest corticosteroid dose, immunosuppressant, or the combination of both, or the presence of recurrences above a dosage threshold.

Exclusion criteria included recent intraocular surgery (three months), prior treatment with another biologic agent (unless at least five half-lives had elapsed), and incomplete ocular examination or tear sample collection at the time of the study. Patient management was conducted by a multidisciplinary team composed of ophthalmologists, rheumatologists, and internists as per individual needs.

### Clinical Characteristics and Disease Evaluation

Patients’ characteristics were recorded including age and sex. Uveitis characteristics, such as laterality and anatomic localization (anterior, intermediate, posterior or panuveitis) were classified per the Standardization of Uveitis Nomenclature Working Group criteria.^15^

Intraocular inflammation severity was classified according to Standardization of Uveitis Nomenclature criteria.^15^ Anterior chamber cells were graded from 0 to 4+ via slit lamp biomicroscopy before mydriatic drops, while vitreous haze was graded from 0 to 4+ through indirect ophthalmoscopy after mydriasis according to the Nussenblatt criteria.^15^^,^^16^

Uveitis was deemed active with at least one active inflammatory chorioretinal or retinal lesion, anterior chamber cell grade of ≥0.5+, or vitreous haze grade of ≥0.5+. Otherwise, it was considered inactive. Patients or eyes with active disease were classified as nonresponders to ADA (NR), while those without intraocular activity were responders (R). Data were managed using the electronic system Research Electronic Data Capture (REDCap) facilitated by Fundación Sociedad Española Farmacia Hospitalaria.^17^

### Tear and Serum Sample Collection

Tear samples were obtained from both eyes using 3-µl Microcaps microcapillary tubes (no. 1-000-0030; Drummond Scientific, Broomall, PA, USA). Collection was done as close as possible to the next ADA administration, no longer than 48 hours before the day of the administration. Unstimulated tears (before clinical assessment or drops instillation and minimizing ocular irritation to avoid reflex tear) were collected via capillarity from the inferior temporal tear meniscus and dispensed into precooled 0.5 mL Protein LoBind tubes (Eppendorf, Madrid, Spain). Patients were instructed not to use artificial tears 24h prior to collection. To prevent keratin contamination, gloves were worn, and special attention was paid to avoid touching the eyelashes or skin with the capillary tip. Samples were kept cold throughout the collection process using the IsoPack/IsoRack set (no. 3880000.160; Eppendorf) to prevent protein degradation, then immediately stored at −20°C.^18^^,^^19^ Patients reported no or minimal discomfort during the procedure. Serum samples were collected to measure ADA through levels using Promonitor ELISA kit as previously described.^20^

### Shotgun Proteomics in Tear Samples and Data Analysis

**Figure 1.**
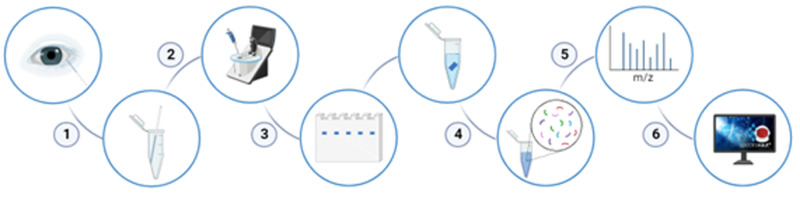
Schematic representation of the steps involved in the proteomic analysis. **(1)** Tear sample collection with microcapillary tubes and storage, **(2)** protein quantification, **(3)** running of protein samples into gel and excision of protein bands, **(4)** in-gel digestion of proteins with trypsin, **(5)** peptide analysis by DIA/SWATH, and **(6)** data analysis.

Total tear protein content was quantified (Fig. 1) using a NanoDrop One spectrophotometer (Thermo Fisher Scientific, Waltham, MA, USA) and then digested to obtain peptides that were injected and analyzed by Data-Independent Acquisition/Sequential Window Acquisition of all Theoretical Mass Spectra (DIA/SWATH) using a Triple TOF 6600 system (Sciex, Framingham, MA, USA). A complete description of this section can be found in the Supplementary Material.

### Statistical Analysis

Continuous variables are depicted as the mean ± standard deviation (SD) or the median with the interquartile range (IQR) if variables were not normally distributed. Categorical variables are shown as frequency and corresponding percentage (%). Comparison of continuous variables was performed with *t*-test or Mann-Whitney U test. Differences in dichotomous variables were assessed with the Fisher's exact test or χ^2^ test as appropriate.

MS data files were processed using Spectronaut Software 16.0 (Biognosys) with library-free directDIA pipeline's standard settings and normalized against the total peptide amount. After normalization, the ratio (NR/R) of protein expression was calculated. DEPs were defined as those with a fold change (ratio) >1.4 or <0.7 with a corresponding *P* value < 0.05. To focus on representative proteins, those present in less than 80% of the samples from the two subgroups were excluded (See Supplementary Fig. S1). Proteomic files are deposited and publicly available in the MassIVE dataset MSV000095892 at MassIVE repository (https://massive.ucsd.edu/).

Protein Interaction network analysis was performed using the STRING database (https://string-db.org/), which enables the creation of a map of known interactions between DEPs identified across groups and provides information about enriched biological processes in which proteins are involved, the subcellular localization and the cellular compartment where they can be found, also known as gene ontology (GO) enrichment analysis. Bubble plot of GO enriched terms was done using SRplot.^21^

The χ^2^ test for trend was used to assess whether there is a linear trend between protein amount of individual DEPs and the proportion of NR/R to ADA. For this analysis, protein amount was ordered from low to high and categorized in quartiles (25th quartile containing lower concentration, 100th quartile higher concentration). To evaluate the potential of DEPs as biomarkers for treatment response, receiver operating characteristic curves were plotted and the area under the curve was calculated. Data were analyzed using GraphPad Prism Software v8. Results were considered statistically significant when *P* < 0.05.

## Results

### Patient Characteristics

Thirty-five patients (58 eyes) were included, with a mean age of 44 years (57% women). Panuveitis was the most common location (34%), followed by anterior uveitis (31%), intermediate and posterior uveitis (both 17%). Bilateral involvement was 74%. NR to ADA accounted for 49% (Table 1).

**Table 1.** Clinical Characteristics of NIU Patients Included in the Study

	All (n = 35)
Age (years), median (IQR)	44.0 (18.0–55.0)
Sex	
Female	20 (57%)
Male	15 (43%)
Duration on ADA treatment (months), median (IQR)	44.0 (18.0–76.0)
ADA posology	
Standard	28 (80%)
Reduced	4 (11.4%)
Intensified	3 (8.6%)
Response to ADA	
NR	17 (48.5%)
R	18 (51.5%)
Serum ADA levels	
Non-responders (µg/mL), median (IQR)	9.74 (8.63–14.2)
Responders (µg/mL), median (IQR)	10.55 (3.96–18.0)
Uveitis location	
Anterior	11 (31.4%)
Intermediate	6 (17.1%)
Posterior	6 (17.1%)
Panuveitis	12 (34.3%)
Laterality	
Unilateral	9 (25.7%)
Bilateral	26 (74.3%)
Uveitis diagnosis	
Recurrent anterior uveitis	11 (31.4%)
Pars planitis	5 (14.3%)
Multifocal choroiditis	2 (5.7%)
Birdshot	3 (8.6%)
Idiopathic panuveitis	7 (20.0%)
Sarcoid	2 (5.7%)
Behçet	1 (2.9%)
Serpiginous choroiditis	1 (2.9%)
Idiopathic intermediate uveitis	1 (2.9%)
Voght-Koyanaghi-Harada	1 (2.9%)
Posterior idiopathic uveitis	1 (2.9%)
Systemic immune-mediated associated disease	
Yes	14 (40%)
No	21 (60%)
Concomitant treatment	
Oral corticoids	
Yes	9 (25.7%)
No	25 (71.4%)
Immunomodulators	
Yes	23 (65.7%)
No	12 (34.3%)

**Figure 2.**
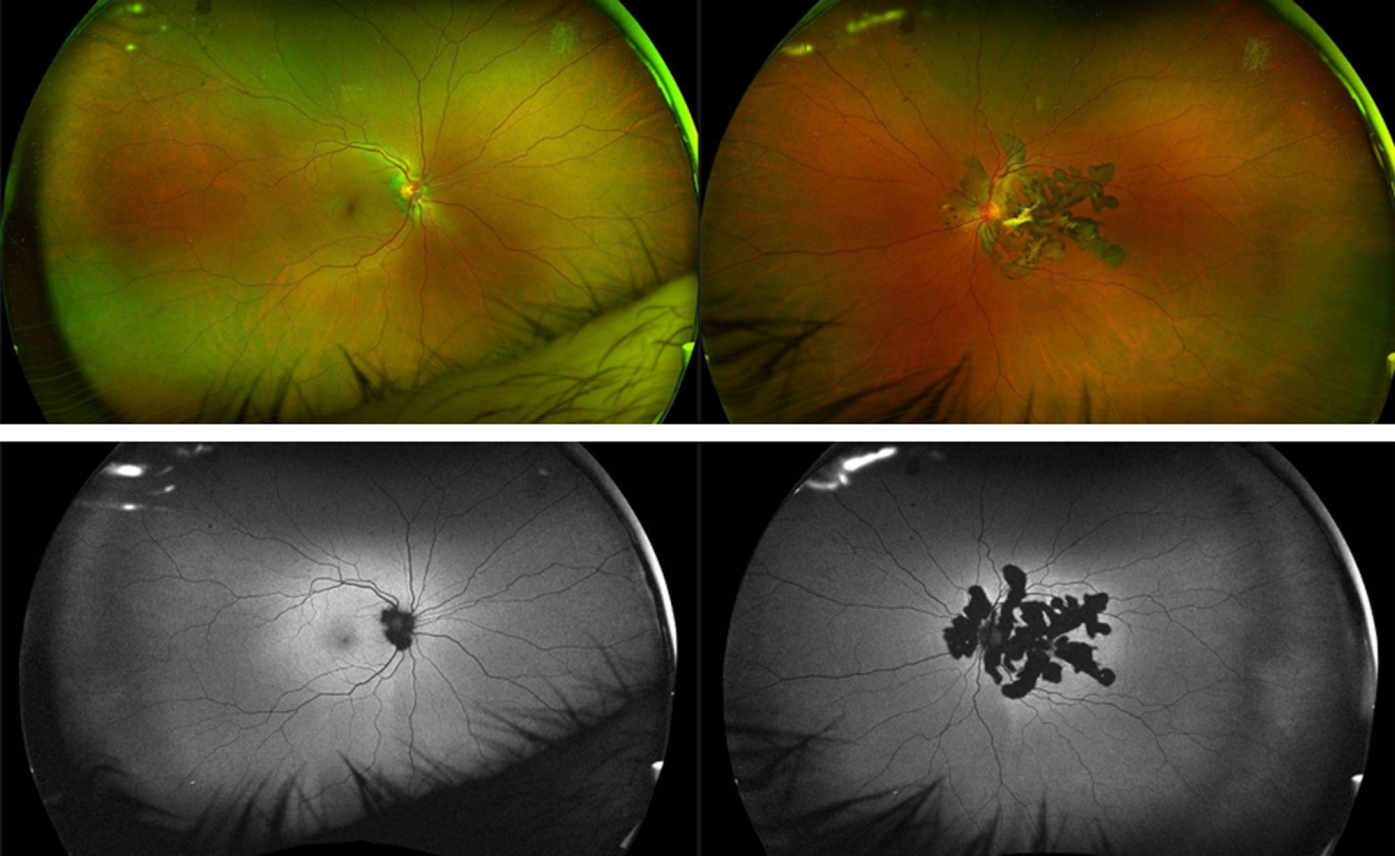
Ocular fundus imaging of right and left eye of a patient with a good response to ADA. Color retinography (*upper image*) and autofluorescence (*lower image*) shown no active lesions despite severe affectation of the posterior pole in left eye. Both anterior chamber cell grade and vitreous haze grade were 0.

Only tear samples of affected eyes were included in the analysis, 32 (55.2%) R and 26 (44.8%) NR (Fig. 2). No differences were observed in protein concentration or total protein between R and NR eyes. Only tear volume was slightly higher in the NR subgroup (See Supplementary Fig. S2).

### Differential Tear Proteomic Profile on Anti-TNF Treatment

**Figure 3.**
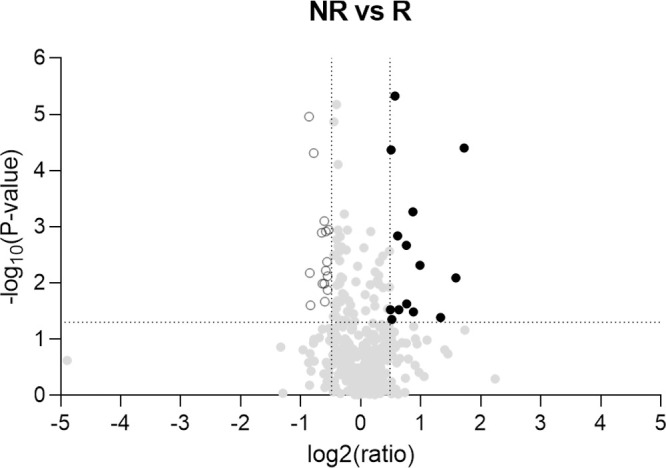
Volcano plots of DEPs in tear samples from NR eyes compared to R eyes to ADA treatment. The ratio of protein expression in NR versus R eyes is displayed in the x-axis as the log^2^ and the −log^10^ of the *P* value obtained from the differential abundance testing is displayed in the y-axis. Therefore proteins displayed at log^2^ values >0 are more abundant in NR eyes and those displayed at values <0 are more abundant in R eyes. Significantly modulated proteins are displayed at −log^10^ (*P* value) >1.3 corresponding to a *P* value = 0.05. Proteins excluded from the Uniprot database or outside our predefined criteria for DEPs are not shown.

In total, 399 proteins were quantified in tear samples (Supplementary Table 1); 29 of those met our predefined DEPs criteria (Table 2). Fourteen proteins were upregulated in NR eyes and 15 were downregulated (Fig. 3).

**Table 2.** Altered Tear Proteins in Patients With NIU Treated With ADA

Accession Number	Abbreviation	Protein Name	Ratio	*P*
Upregulated proteins in nonresponders				
P59665; P59666	DEF1; DEF3	Neutrophil defensin 1; Neutrophil defensin 3	3.30	0.00004
P05090	APOD	Apolipoprotein D	3.00	0.008
P02675	FIBB	Fibrinogen beta chain	2.52	0.04
Q16378	PROL4	Proline-rich protein 4	1.98	0.005
Q14515	SPRL1	SPARC-like protein 1	1.84	0.03
P0C0L4	C4A	Complement C4-A	1.83	0.0005
P62805	H4	Histone H4	1.70	0.02
P05109	S100A8	Protein S100-A8	1.69	0.002
Q8WXG9	AGRV1	Adhesion G-protein coupled receptor V1	1.55	0.03
O75556	SG2A1	Mammaglobin-B	1.53	0.001
P43251	BTD	Biotinidase	1.49	0.000005
O95968	SG1D1	Secretoglobin family 1D member 1	1.43	0.04
Q9GZZ8	LACRT	Extracellular glycoprotein lacritin	1.42	0.00004
P06702	S100A9	Protein S100-A9	1.41	0.03
Downregulated proteins in nonresponders				
Q16651	PRSS8	Prostasin	0.69	0.001
Q13228	SBP1	Methanethiol oxidase	0.68	0.01
P26447	S100A4	Protein S100-A4	0.68	0.008
P09211	GSTP1	Glutathione S-transferase P	0.68	0.004
Q06830	PRDX1	Peroxiredoxin-1	0.67	0.001
P04792	HSPB1	Heat shock protein beta-1	0.67	0.006
Q9GZN4	BSSP4	Brain-specific serine protease 4	0.66	0.02
P01037	CST1	Cystatin-SN	0.66	0.0008
P68104; Q5VTE0	EF1A1; EF1A3	Elongation factor 1-alpha 1; Putative elongation factor 1-alpha-like 3	0.66	0.01
P35527	K1C9	Keratin, type I cytoskeletal 9	0.64	0.01
P07437	TBB5	Tubulin beta chain	0.64	0.001
P00738	HP	Haptoglobin	0.58	0.00005
O95477	ABCA1	Phospholipid-transporting ATPase ABCA1	0.56	0.03
Q6MZM9	PRR27	Proline-rich protein 27	0.56	0.007
P00352	ALDH1A1	Aldehyde dehydrogenase 1A1	0.55	0.00001

Proteins are listed in decreasing order of ratio calculated by dividing the amount of protein quantified in NR eyes by that quantified in R eyes. Ratios >1 indicate protein overexpression in the NR subgroup, whereas ratios <1 indicate lower protein expression in the NR subgroup, *i.e.* proteins are overexpressed in the R subgroup. Accession numbers from UniProt database (https://www.uniprot.org/). DEF1;DEF3 and EF1A1;EF1A3 could not be unambiguously identified by unique peptides likely due to their high homology (sequences differ by one amino acid), assessed with the freely available resource SIM - Alignment Tool for Protein Sequences (https://web.expasy.org/sim/). Proteins excluded from the Uniprot database, such as most non-germline immunoglobulins and T-cell receptors, are not shown.

### Altered Proteins in NRs Have Immunomodulatory Roles

**Figure 4.**
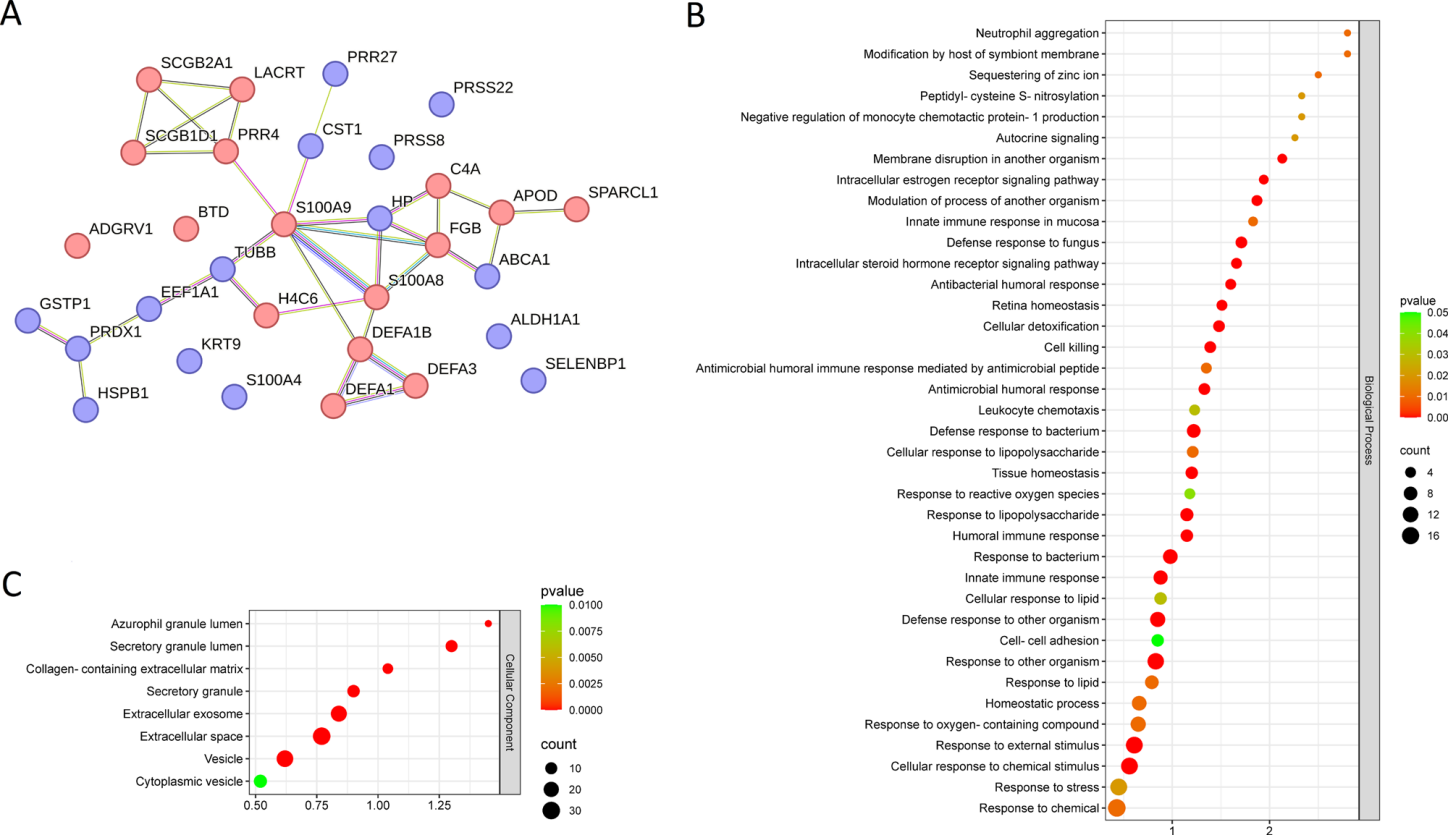
STRING Interaction Network and GO analysis of representative DEPs in the nonresponder and the responder eye subgroups. (**A**) Interaction network analysis. Proteins are depicted as nodes (*circles*), and *lines* represent the interactions between any two proteins either direct (physical) or indirect (functional) in nature. Upregulated proteins in NR eyes are shown in *red*, downregulated proteins are shown in blue. (**B** and (**C**) GO enrichment analysis. The bubble plot displays the biological processes (**B**) in which DEPs participate (those upregulated and downregulated in NR) at the top, and the cellular components (**C**) where they are located.

The STRING database for protein-protein interaction analysis (Fig. 4A) revealed one principal network of interacting proteins containing most DEPs. GO enrichment analysis showed that modulated proteins were involved in biological processes mainly related to immunomodulatory and antimicrobial activity, redox balance, and retina homeostasis (Fig. 4B) and secreted into the extracellular space by vesicles (Fig. 4C).

Proteins with protective activity against reactive oxygen species (ROS) and other reactive toxic substances were decreased in the NR group, namely peroxiredoxin-1 (PRDX1), glutathione S-transferase P (GSTP1), aldehyde dehydrogenase 1 A1 (ALDH1A1) and haptoglobin (HP). Cystatin-SN (CST1) and ATP-binding cassette transporter (ABCA1), involved in tissue homeostasis, were also decreased in NR. Upregulated proteins in NR with a role in immunomodulatory or antimicrobial processes included protein S100A8 (S100A8), protein S100A9 (S100A9), α-defensins (DEF1;DEF3), anaphylatoxin C4a (C4A), and fibrinogen β (FIBB), implicated in pro-inflammatory processes.

### Potential Biomarkers for Treatment Response

**Figure 5.**
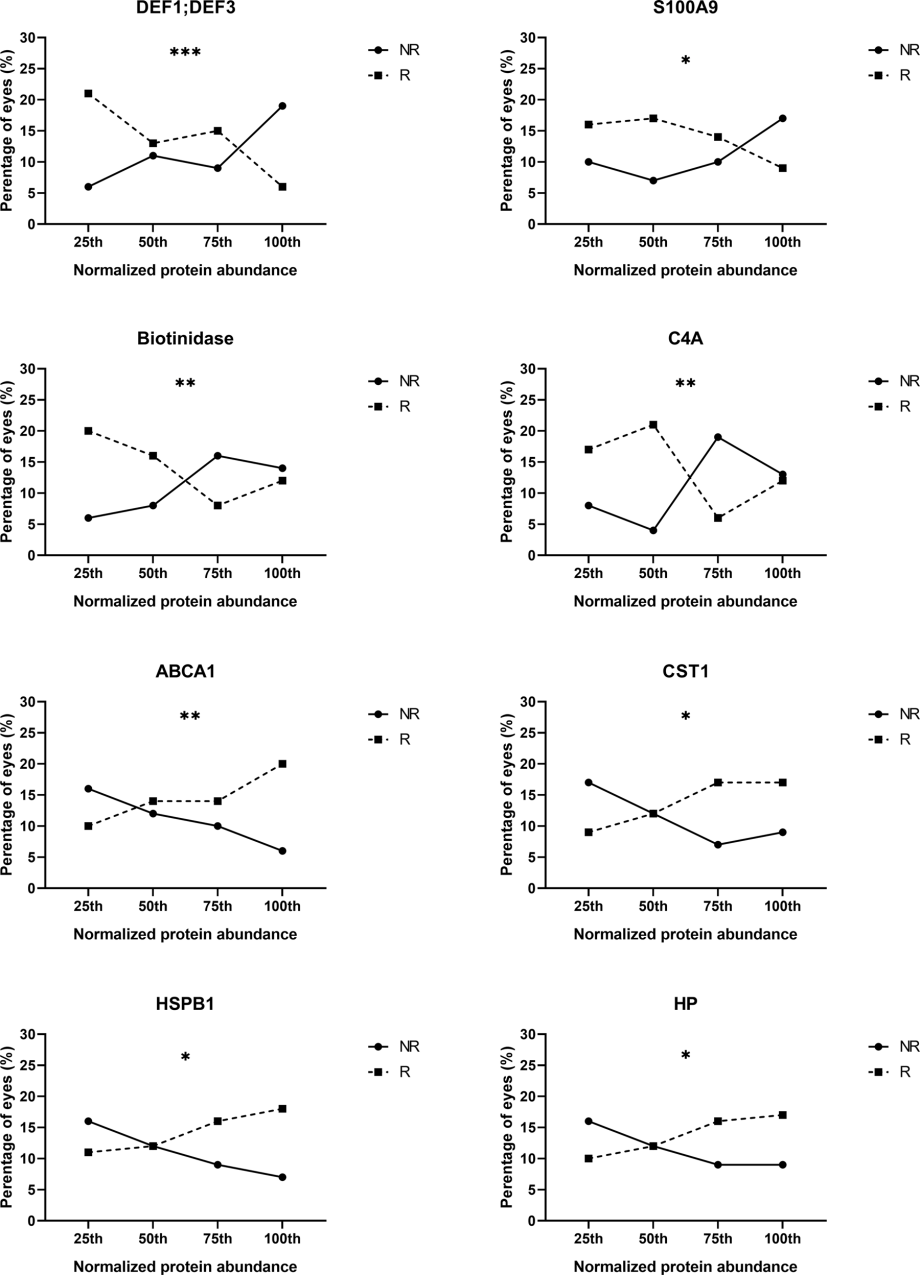
Relationship between the prevalence of NR and R eyes with protein abundance expressed in increasing order from the lowest (25th) to the highest (75th) quartile. Statistical analysis was done using χ^2^ test for trend (*P < 0.05, **P < 0.01, ***P < 0.001).

**Figure 6.**
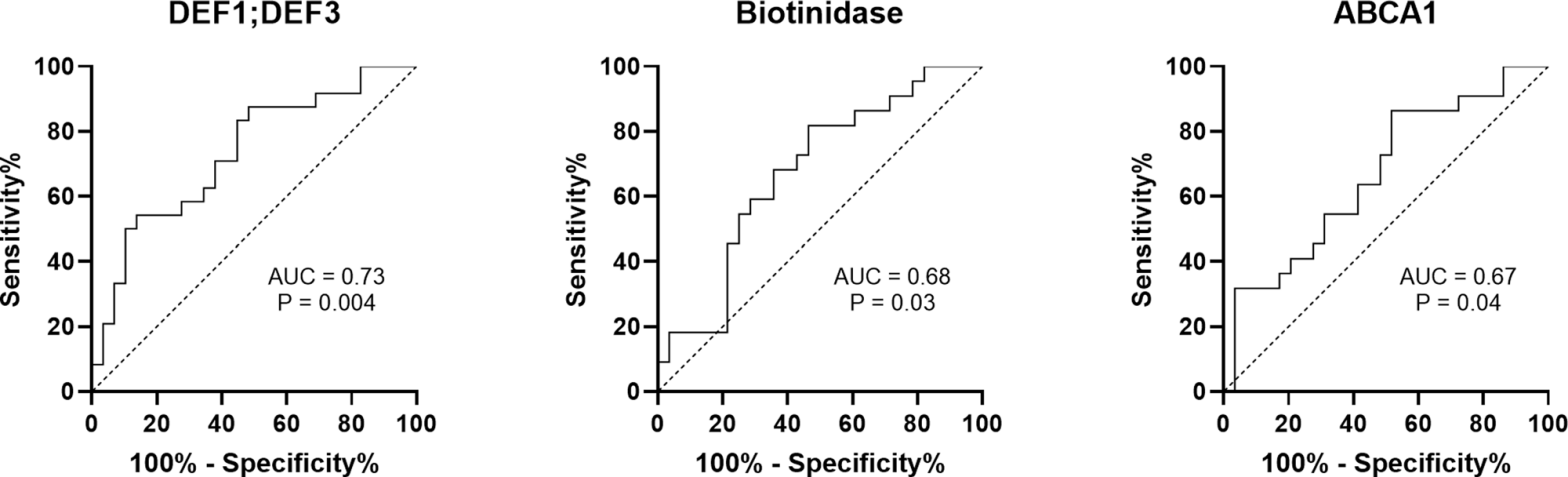
Potential tear protein biomarkers for treatment response to ADA based on receiver operating characteristic curve analysis.

These 29 identified DEPs were further investigated as potential as biomarkers for treatment response. Significant relationships between protein abundance and frequency of response to ADA were found for eight proteins (Fig. 5). A trend toward a higher percentage of NR eyes was found with increasing concentrations of DEF1;DEF3, S100A9, biotinidase, and C4A and with decreasing concentrations of ABCA1, CST1, HP and HSPB1, in line with their expression in NR (Supplementary Fig. S3). Assessment of the ability of these eight proteins to discriminate between R and NR revealed that DEF1;DEF3, biotinidase, and ABCA1 were promising as potential treatment response biomarkers, with moderate area under the curve values of 0.73, 0.68, and 0.67 (Fig. 6), respectively.

## Discussion

This is the first study pointing toward neutrophil hyperactivation as the underlying mechanism behind the persistent intraocular inflammation in patients with NIU despite anti-TNFα treatment, regardless of uveitis type and associated systemic disease, by using high-throughput label-free quantitative proteomics in tear samples. Our results suggested neutrophil-related innate immunity imbalance as the cause of inflammation seen in NR patients, which could inform decisions regarding biological therapy changes when dealing with multidrug-resistant uveitis. In addition, three of the altered proteins, DEF1;DEF3, biotinidase, and ABCA1, were identified as promising biomarkers of treatment response.

Reduced expression of antioxidant proteins suggests compromised antioxidant activity. PRDX1 converts highly reactive hydrogen peroxide and hydroperoxide compounds into less toxic compounds.^22^ GSTP1 reacts with potentially toxic electrophilic compounds.^23^ ALDH1A1 functions as a retinal dehydrogenase preventing the formation of cytotoxic protein adducts by oxidizing reactive aldehydes.^24^ HP scavenges free hemoglobin preventing its oxidative damage.^25^ Impairment of antioxidant defenses can lead to ROS-mediated cell oxidative damage, known to induce sustained inflammation and tissue injury.^26^ In fact, an enrichment of pathways related to responses to oxygen-containing compounds was detected in NR, and neutrophils are known to produce large amounts of ROS as a defense against microbial infections. CST1 expression was also downregulated in NR, which has been proposed to have a role in eye homeostasis through its immunomodulatory activity and the inhibition of exogenous proteases.^27^

One of the main sources of ROS in neutrophils is NADPH-oxidase. This enzyme is activated by the S100A8/S100A9 complex, also known as calprotectin,^28^ which was upregulated in NR, suggesting that inflammation could be due to calprotectin-mediated NADPH-oxidase-dependent ROS production. Calprotectin can also acts as damage-associated molecular pattern molecule and promote inflammation upon interaction with toll-like receptor 4 (TRL4) or with receptor for advanced glycation end-products, leading to recruitment and activation of neutrophils and other immune cells.^29^^–^^31^ In patients with NIU, S100A8/S100A9 levels have been correlated with the severity of intraocular inflammation.^32^^–^^34^ Additionally, S100A8/S100A9 can upregulate IL-6 production and activate the complement system, creating a vicious cycle of chronic inflammation-mediated tissue destruction in immune-mediated diseases.^35^

Neutrophil recruitment, survival, ROS production and proinflammatory cytokine expression are modulated by fibrinogen present at sites of inflammation.^36^^–^^38^ A link between fibrinogen alteration and neutrophil NADPH-oxidase ROS production has been proposed in the pathogenesis of Behçet disease (BD),^39^ which may be common to other NIU forms, as we observed increased FIBB expression in >80% of NR, including patients without BD.

These and other modulated proteins related to neutrophil effector functions indicate neutrophil recruitment and activation in NR. The α-defensins (α-DEFs) such as DEF1 and DEF3 are antimicrobial proteins secreted by activated neutrophils that induce TNF-α and interferon γ (INF-γ)–mediated inflammation^40^ and have emerged as promising biomarkers of treatment response in this study. Another role of DEF-1 is the inhibition of the complement classical and lectin pathways.^41^ However, our observation of increased C4A expression suggests these pathways are activated. C4A expression has been related to gene copy number variations and higher IL-6 expression in BD,^42^ involved in neutrophil trafficking, activation, enhancing effector function and survival.^43^^,^^44^ C4A also inhibits monocyte chemotaxis,^45^ aligning with the enriched GO term for negative regulation of monocyte chemotactic protein-1 (MCP-1) production, a key regulator of monocyte recruitment. Previously, predominant neutrophil rather than monocyte infiltration in rheumatoid arthritis (RA) synovial fluid was attributed to C4A.^46^ Additionally, neutrophil recruitment to perivascular areas of the retina has been described in autoimmune uveitis models.^47^^,^^48^ Similarly, our results suggest neutrophils are the infiltrated cells driving inflammation in the eyes of NR patients; however, we have not confirmed this in our patients.

Downregulation of ABCA1 is another evidence of neutrophil activation. This protein, involved in high density lipoprotein formation through cholesterol transport,^49^ can attenuate neutrophil-induced inflammation in vivo and reduce cytokine production in vitro (IL-1β, IL-6, TNF-α).^49^^–^^52^ Remarkably, ABCA1 was identified as a potential biomarker in our study, whereas IL-1β, IL-6, and TNF-α were not detected in our samples.

Overall, our findings point to a hyperactivation of neutrophils as the underlying mechanism behind the persistent inflammation in NIU patients unresponsive to ADA, regardless of uveitis type and associated systemic disease. Increased neutrophil infiltration, hyperactivated phenotype and aberrant responses after stimulation have already been reported in autoimmune uveitis models.^47^^,^^53^^–^^55^ Moreover, hyperactivated neutrophils have been implicated in the pathogenesis of BD,^56^ juvenile idiopathic arthritis (JIA),^57^ RA,^58^ and other chronic inflammatory and autoimmune diseases.^59^ Importantly, this hyperactivation may be a permanent rather than transient feature, because it is still observed in stages of clinical remission,^60^ linking genetics to this hyperactivated phenotype.^61^ Because anti-TNF-α treatment has been shown to partially reverse exacerbated neutrophil function in vitro,^62^ it is possible that this effect occurs to a lesser extent in patients with NIU unresponsive to ADA, maybe because of subtherapeutic drug levels. Consequently, hyperactivated neutrophils may drive chronic inflammation and tissue damage, creating a destructive cycle refractory to anti-TNF therapy. However, addressing whether insufficient ADA levels contribute to this mechanism remains challenging because of the lack of a defined a therapeutic range for ADA in NIU.^7^

Considering that our findings suggest neutrophil involvement in autoimmune diseases, we propose that the therapeutic target of neutrophil effector functions may be beneficial for NIU patients failing anti-TNFα therapy. This underscores the importance of evidence-based approaches over subjective clinical decision-making to improve patient management. One evident option is targeting proinflammatory cytokines or its receptors, such as IL-6 receptor, whose blockade with tocilizumab has shown promise in NIU.^63^ Targeting downstream cytokine signaling related to neutrophil activation with Janus kinase proteins inhibitors^64^ like tofacitinib (NCT03580343), baricitinib (NCT04088409), and filgotinib (NCT03207815) is another treatment strategy that is currently being evaluated in NIU. Inhibiting the complement cascade with eculizumab or ravulizumab could also be beneficial, although their efficacy in NIU remains unexplored.^65^ Interfering with neutrophil migration/extravasation represents an interesting therapeutic approach, for example through inhibition of CXCR1 and CXCR2,^53^ receptors for the neutrophil chemoattractant IL-8 or overexpression of endomucin, a glycoprotein expressed by endothelial cells that has been shown to prevent neutrophil recruitment into sites of inflammation.^66^ Although the possibilities are broad,^67^ repurposed drugs are clearly the fastest way to have safe and effective treatments available at a lower cost.^68^

We acknowledge that cross-sectional design, relatively small sample size and heterogeneous ADA treatment duration are some limitations of this study. However, in the largest collection of ADA-treated patients to date, we have identified a network of modulated proteins involved in intraocular inflammation common to different uveitis types. These results need further replication, ideally in prospective studies involving NIU patients naïve to ADA, to assess the definitive value of DEF, biotinidase and ABCA1 as biomarkers in clinical practice. Nonetheless, including treatment-naïve patients remains challenging because of the disease's low prevalence.

## Conclusions

Our study is the first to use shotgun proteomics to identify modulated proteins in the tears that drive intraocular inflammation, shedding light on mechanisms underlying ADA treatment failure across various NIU types. Neutrophil hyperactivation and redox imbalance may account for the persistent eye inflammation, as evidenced by upregulation of proteins related to neutrophil recruiting, activation and proinflammatory effector functions and downregulation of proteins with antioxidant and anti-inflammatory activity in NR. DEF-1,3, biotinidase and ABCA1 were identified as potential tear biomarkers of treatment response. Additionally, IL-6, Janus kinase proteins, and the complement cascade pathways were identified as possible alternative therapeutic targets. Our work demonstrates that DIA/SWATH proteomics enables protein profiling in small and complex samples like tears, thus achieving a deeper molecular characterization of ophthalmic pathologies. This suggests the potential value of tears as a source of biomarkers and novel therapeutic targets for personalized medicine. Our findings require further validation in larger cohorts of patients to better understand the processes involved in non-response to anti-TNFα.

## Supplementary Material

Supplement 1
