# Supplementary material for: Potential Prognostic Protein Biomarkers in Tears From Noninfectious Uveitis Patients Under Biologic Treatment as a Prelude to Personalized Medicine
Source: Invest Ophthalmol Vis Sci. 2024 Nov 14;65(13):29. doi: 10.1167/iovs.65.13.29 (PMC11572757; doi:10.1167/iovs.65.13.29)
Supplement: Supplement 1 [file iovs-65-13-29_s001.pdf]

SUPPLEMENTARY MATERIAL

Tear Protein analysis by LC-MS/MS

Protein content was directly quantified from tears at 280 nm using 1-2 uL of sample. After measurement, samples were recovered to the corresponding tube. Protein was loaded on a 10% SDS-PAGE, bands were revealed using Sypro-Ruby fluorescent staining (Lonza, Switzerland), excised and processed for in-gel digestion with trypsin. Peptides were extracted with 60% acetonitrile in 0.5% formic acid by 3 rounds of 20-minute incubation, concentrated by vacuum evaporation and stored at −20 °C.

LC-MS/MS analysis

Peptides were separated by Reverse Phase Chromatography with gradient elution using a micro liquid chromatography (LC) system (Eksigent Technologies nanoLC 400, SCIEX) coupled to a hybrid quadrupole-TOF Triple mass spectrometer TOF 6600 (SCIEX) for data acquisition.

Samples (4 µl) were injected at 10 µL/min into a Chrom XP C18 (150 mm × 0.30 mm) analytical column with 3 µm particle size and 120 Å pore size (Eksigen, Dublin, CA, USA), switched on-line with the YMC-TRIART C18 (YMC Technologies, Teknokroma) trap column with similar particle and pore size. Peptides were eluted at 5 µL/min using the following gradient: from 5% to 95% of phase B for 30 min, 5 min at 90% B and finally 5 min at 5% B for column equilibration, for a total run time of 40 min (phase A 0.1% formic acid in water, phase B 0.1% formic acid in 100% acetonitrile).

Source and interface conditions for data acquisition were as follows: IonSpray voltage floating 5500 V, Curtain Gas at 25, Collision Energy 10 and ion source gas 1 (GS1) at 25. Instrument was operated with Analyst TF 1.7.1 software (SCIEX, USA).

Protein quantification by SWATH (Sequential Window Acquisition of all Theoretical Mass Spectra)

SWATH–MS was acquired in data-independent acquisition (DIA) mode using 3 technical replicates per sample. The method consisted of repeating the following cycle: acquisition of 65 TOF MS/MS scans (400 to 1500 m/z, high sensitivity mode, 50 ms acquisition time) of overlapping sequential precursor isolation windows of variable width (1 m/z overlap) covering the 400 to 1250 m/z mass range with a previous TOF MS scan (400 to 1500 m/z, 50 ms acquisition time) for each cycle of 6.3 s total time. The instrument was automatically calibrated every 4 hours using as external calibrant tryptic peptides from SWATH Acquisition Performance Kit PepCalMix (Sciex).

MS data files were processed using Spectronaut Software 16.0 (Biognosys) with library-free directDIA default settings (Trypsin/P, 2 missed cleavages). For DIA Identification: Precursor Qvalue Cutoff: 0,01; Precursor PEP Cutoff: 0,2; Protein Qvalue Cutoff (Experiment): 0,01; Protein Qvalue Cutoff (Run): 0,05 and Protein PEP Cutoff: 0,75. Protein and Peptide FDR: 0,01. Search criteria included carbamidomethyl (C) as a fixed modification and acetyl (Protein N-term) and oxidation (M) as variable modifications. Single Hit Proteins and Predicted Fragment Scores were not excluded. Duplicate Assays were excluded. An automatic cross run normalization strategy was performed with interference correction. An unpaired t-test was used to evaluate differential abundance without assuming equal variances between groups.

SUPPLEMENTARY FIGURES

Supplementary Figure 1. Examples of protein quantification results obtained in the proteomic analysis to distinguish between representative and non-representative proteins. Proteins detected and quantified in 80% of the samples or more from each subgroup (A) are considered as representative, otherwise are considered as non-representative (B) and excluded from further analysis. Each dot represents one tear sample. Dots plotted in the yellow and red area belong to the subgroup of responder and non-responder eyes, respectively.

A

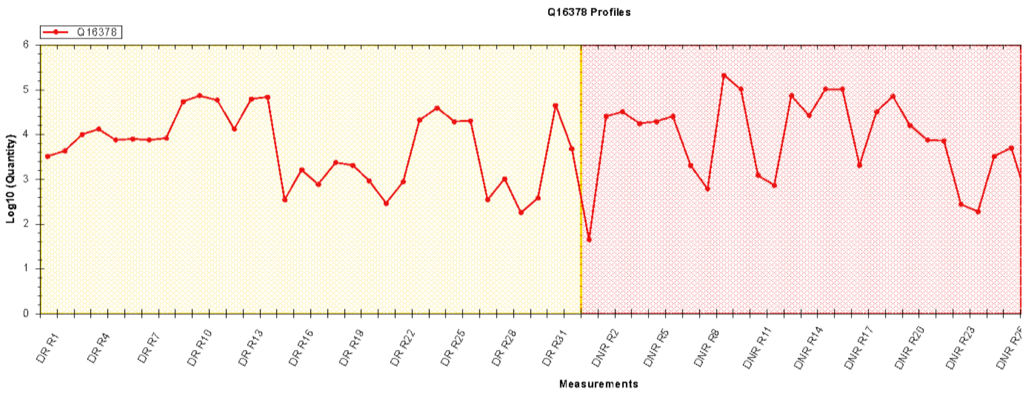

B

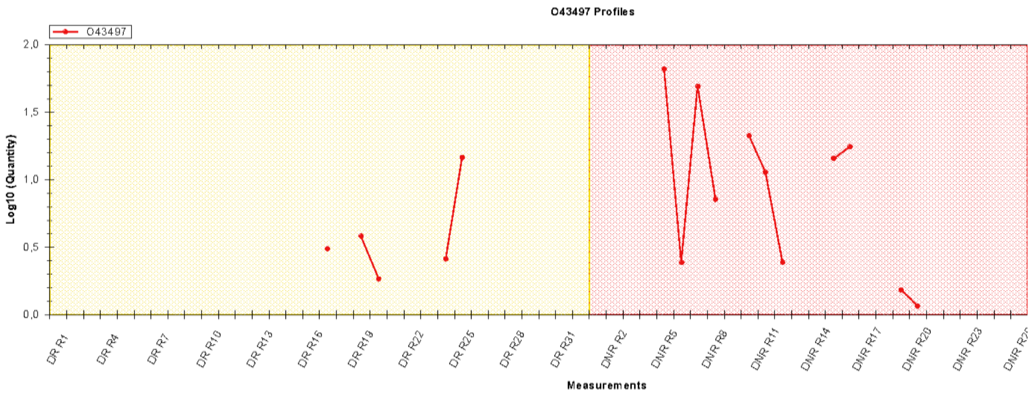

Supplementary Figure 2. Comparison of the characteristics of tear samples obtained from NIU patients stratified according to their response to anti-TNFα.

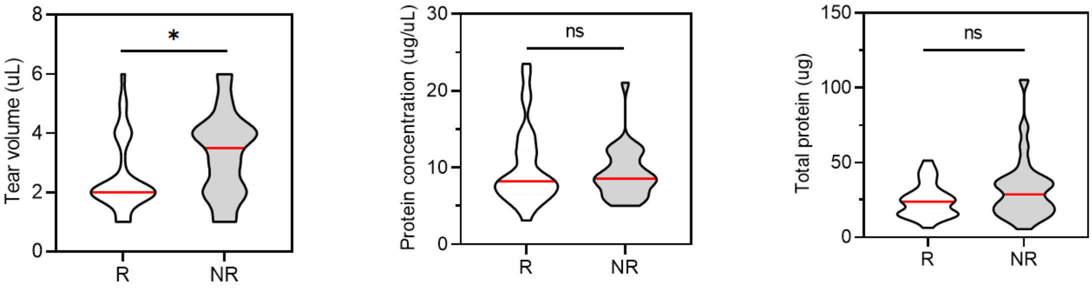

Supplementary Figure 3. Comparison of tear protein abundance expressed as normalized protein quantity between patients with NIU responders and non-responders to anti-TNFα for the 8 potential biomarkers. Statistical analysis was done using Mann-Whitney test (* $p < 0.05$, ** $p < 0.01$). NR = non-responder, R = responder.

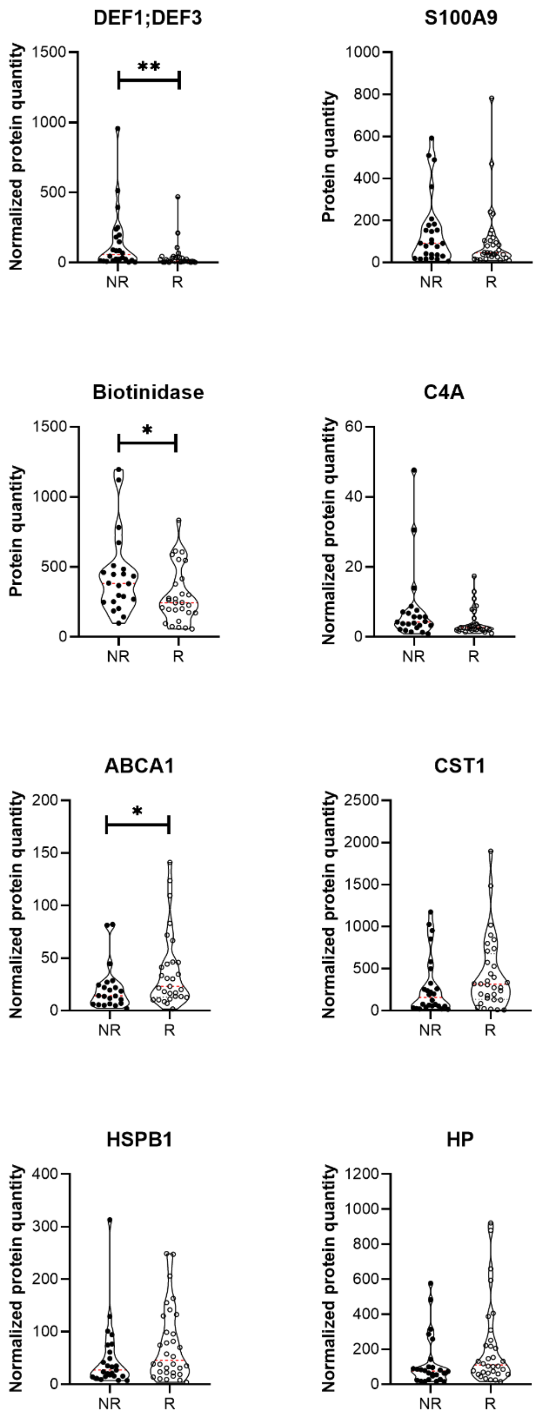

SUPPLEMENTARY TABLES

Supplementary Table 1. List of identified proteins in tear samples from patients with NIU treated with ADA.

ProteinAccessions	ProteinDescriptions	ProteinNames	AvgLog2Quantity	Pvalue
A0A075B6H7	Probable non-functional immunoglobulin kappa variable 3-7	KV37_HUMAN	6,600000	3,197633E-06
A0A075B6H9	Immunoglobulin lambda variable 4-69	LV469_HUMAN	5,834371	2,763652E-08
A0A075B6I1	Immunoglobulin lambda variable 4-60	LV460_HUMAN	3,336177	7,476273E-09
A0A075B6I9	Immunoglobulin lambda variable 7-46	LV746_HUMAN	3,516248	7,476273E-09
A0A075B6J9	Immunoglobulin lambda variable 2-18	LV218_HUMAN	3,769496	2,559754E-06
A0A075B6K4	Immunoglobulin lambda variable 3-10	LV310_HUMAN	5,992885	2,121326E-06
A0A075B6K5	Immunoglobulin lambda variable 3-9	LV39_HUMAN	5,140868	1,269610E-07
A0A075B6P5	Immunoglobulin kappa variable 2-28	KV228_HUMAN	8,992151	7,720822E-08
A0A075B6R9	Probable non-functional immunoglobulin kappa variable 2D-24	KVD24_HUMAN	6,979337	2,009343E-07
A0A087WSX0	Immunoglobulin lambda variable 5-45	LV545_HUMAN	1,330310	1,663578E-05
A0A087WSY6	Immunoglobulin kappa variable 3D-15	KVD15_HUMAN	7,010000	7,476273E-09
A0A0A0MRZ8	Immunoglobulin kappa variable 3D-11	KVD11_HUMAN	9,641004	1,066471E-07
A0A0A0MS14	Immunoglobulin heavy variable 1-45	HV145_HUMAN	3,896800	6,935247E-07
A0A0A0MS15	Immunoglobulin heavy variable 3-49	HV349_HUMAN	6,914847	4,195220E-06
A0A0A0MT36	Immunoglobulin kappa variable 6D-21	KVD21_HUMAN	2,100000	4,404472E-05
A0A0C4DH26	Probable non-functional immunoglobulin kappa variable 6D-41	KVD41_HUMAN	2,100000	4,404472E-05
A0A0B4J1U7	Immunoglobulin heavy variable 6-1	HV601_HUMAN	5,674119	7,476273E-09
A0A0B4J1V0	Immunoglobulin heavy variable 3-15	HV315_HUMAN	9,925549	4,285954E-08
A0A0B4J1V2	Immunoglobulin heavy variable 2-26	HV226_HUMAN	2,400000	1,607130E-06
A0A0B4J1X5	Immunoglobulin heavy variable 3-74	HV374_HUMAN	10,293233	9,730754E-08
A0A0B4J1Y8	Immunoglobulin lambda variable 9-49	LV949_HUMAN	4,550000	1,241989E-06
A0A0B4J2D9	Immunoglobulin kappa variable 1D-13	KVD13_HUMAN	5,629756	1,365980E-04
A0A0C4DH24	Immunoglobulin kappa variable 6-21	KV621_HUMAN	3,333865	1,769025E-05

A0A0C4DH25	Immunoglobulin kappa variable 3D-20	KVD20_HUMAN	7,943569	6,511999E-08
A0A0C4DH35	Probable non-functional immunoglobulin heavy variable 3-35	HV335_HUMAN	5,671631	3,216194E-06
A0A0C4DH38	Immunoglobulin heavy variable 5-51	HV551_HUMAN	5,815617	2,056325E-05
A0A0C4DH41	Immunoglobulin heavy variable 4-61	HV461_HUMAN	9,290000	2,105514E-07
P01824	Immunoglobulin heavy variable 4-39	HV439_HUMAN	9,290000	2,105514E-07
P01825	Immunoglobulin heavy variable 4-59	HV459_HUMAN	9,290000	2,105514E-07
P06331	Immunoglobulin heavy variable 4-34	HV434_HUMAN	9,290000	2,105514E-07
P0DP06	Immunoglobulin heavy variable 4-30-4	HVD34_HUMAN	9,290000	2,105514E-07
P0DP08	Immunoglobulin heavy variable 4-38-2	HVD82_HUMAN	9,290000	2,105514E-07
A0A0C4DH43	Immunoglobulin heavy variable 2-70D	HV70D_HUMAN	7,133022	1,087525E-07
A0A0C4DH67	Immunoglobulin kappa variable 1-8	KV108_HUMAN	7,011555	2,324013E-08
A0A0C4DH69	Immunoglobulin kappa variable 1-9	KV109_HUMAN	7,011555	2,324013E-08
A0A0J9YX35	Immunoglobulin heavy variable 3-64D	HV64D_HUMAN	3,779191	7,476273E-09
A0A0J9YXX1	Immunoglobulin heavy variable 5-10-1	HV5X1_HUMAN	5,285408	1,877838E-06
A0M8Q6	Immunoglobulin lambda constant 7	IGLC7_HUMAN	1,041101	4,545837E-08
A6NCI8	Uncharacterized protein C2orf78	CB078_HUMAN	12,297409	3,848228E-05
A8MXE2	Beta-1,3-galactosyltransferase 9	B3GT9_HUMAN	2,279003	1,207894E-06
B0FP48	Uroplakin-3b-like protein 1	UPK3L_HUMAN	2,952985	1,216530E-08
E5RIL1	Uroplakin-3b-like protein 2	UPKL2_HUMAN	2,952985	1,216530E-08
O00391	Sulfhydryl oxidase 1	QSOX1_HUMAN	4,282944	8,027348E-09
O00584	Ribonuclease T2	RNT2_HUMAN	4,072016	7,476273E-09
O00744	Protein Wnt-10b	WN10B_HUMAN	2,504701	1,399650E-04
O14773	Tripeptidyl-peptidase 1	TPP1_HUMAN	3,805281	7,949578E-08
O15511	Actin-related protein 2/3 complex subunit 5	ARPC5_HUMAN	1,938806	1,433433E-05
O43405	Cochlin	COCH_HUMAN	1,955693	5,982589E-08
O43426	Synaptojanin-1	SYNJ1_HUMAN	3,913195	1,770338E-05
O43490	Prominin-1	PROM1_HUMAN	2,665100	3,000818E-06
O43653	Prostate stem cell antigen	PSCA_HUMAN	6,691610	2,667335E-07

O43707	Alpha-actinin-4	ACTN4_HUMAN	2,472660	4,484032E-08
P12814	Alpha-actinin-1	ACTN1_HUMAN	2,472660	4,484032E-08
O43852	Calumenin	CALU_HUMAN	2,807411	1,074213E-07
O60641	Clathrin coat assembly protein AP180	AP180_HUMAN	9,474188	9,411139E-05
O60814	Histone H2B type 1-K	H2B1K_HUMAN	4,376062	4,995346E-08
P57053	Histone H2B type F-S	H2BFS_HUMAN	4,376062	4,995346E-08
P58876	Histone H2B type 1-D	H2B1D_HUMAN	4,376062	4,995346E-08
P62807	Histone H2B type 1-C/E/F/G/I	H2B1C_HUMAN	4,376062	4,995346E-08
Q5QNW6	Histone H2B type 2-F	H2B2F_HUMAN	4,376062	4,995346E-08
Q93079	Histone H2B type 1-H	H2B1H_HUMAN	4,376062	4,995346E-08
Q99877	Histone H2B type 1-N	H2B1N_HUMAN	4,376062	4,995346E-08
Q99879	Histone H2B type 1-M	H2B1M_HUMAN	4,376062	4,995346E-08
Q99880	Histone H2B type 1-L	H2B1L_HUMAN	4,376062	4,995346E-08
O75083	WD repeat-containing protein 1	WDR1_HUMAN	1,712113	7,493970E-05
O75503	Ceroid-lipofuscinosis neuronal protein 5	CLN5_HUMAN	2,868410	1,343524E-05
O75556	Mammaglobin-B	SG2A1_HUMAN	12,110000	2,141769E-08
O75787	Renin receptor	RENR_HUMAN	2,535224	3,248526E-07
O75888	Tumor necrosis factor ligand superfamily member 13	TNF13_HUMAN	3,200000	3,271358E-06
O94760	N(G),N(G)-dimethylarginine dimethylaminohydrolase 1	DDAH1_HUMAN	3,024866	1,966360E-04
O95436	Sodium-dependent phosphate transport protein 2B	NPT2B_HUMAN	2,168785	3,590396E-08
O95477	Phospholipid-transporting ATPase ABCA1	ABCA1_HUMAN	4,305701	7,476273E-09
O95968	Secretoglobin family 1D member 1	SG1D1_HUMAN	12,828019	8,292309E-09
P00338	L-lactate dehydrogenase A chain	LDHA_HUMAN	3,870000	3,596187E-08
P00352	Aldehyde dehydrogenase 1A1	AL1A1_HUMAN	3,346137	5,565295E-08
P00450	Ceruloplasmin	CERU_HUMAN	5,304877	7,476273E-09
P00558	Phosphoglycerate kinase 1	PGK1_HUMAN	2,547784	1,906506E-07
P00709	Alpha-lactalbumin	LALBA_HUMAN	3,999959	4,119860E-04
P00738	Haptoglobin	HPT_HUMAN	6,730605	7,476273E-09

P00751	Complement factor B	CFAB_HUMAN	4,090000	2,054448E-08
P01008	Antithrombin-III	ANT3_HUMAN	3,136720	2,635218E-08
P01009	Alpha-1-antitrypsin	A1AT_HUMAN	6,738092	7,476273E-09
P01011	Alpha-1-antichymotrypsin	AACT_HUMAN	7,399982	7,476273E-09
P01024	Complement C3	CO3_HUMAN	8,347504	7,476273E-09
P01033	Metalloproteinase inhibitor 1	TIMP1_HUMAN	5,281990	1,333915E-08
P01034	Cystatin-C	CYTC_HUMAN	7,060000	2,297118E-08
P01036	Cystatin-S	CYTS_HUMAN	13,196314	1,580773E-08
P01037	Cystatin-SN	CYTN_HUMAN	7,605004	1,129938E-07
P01040	Cystatin-A	CYTA_HUMAN	2,990837	1,644300E-04
P01042	Kininogen-1	KNG1_HUMAN	1,946066	6,775555E-07
P01591	Immunoglobulin J chain	IGJ_HUMAN	12,932337	5,108153E-08
P01593	Immunoglobulin kappa variable 1D-33	KVD33_HUMAN	7,505535	9,747537E-09
P01597	Immunoglobulin kappa variable 1-39	KV139_HUMAN	5,637258	7,477692E-08
P01599	Immunoglobulin kappa variable 1-17	KV117_HUMAN	3,815738	7,476273E-09
P01619	Immunoglobulin kappa variable 3-20	KV320_HUMAN	9,974205	1,346507E-08
P01624	Immunoglobulin kappa variable 3-15	KV315_HUMAN	10,369142	1,407462E-07
P01700	Immunoglobulin lambda variable 1-47	LV147_HUMAN	7,622006	9,597574E-09
P01701	Immunoglobulin lambda variable 1-51	LV151_HUMAN	6,906071	2,616632E-08
P01703	Immunoglobulin lambda variable 1-40	LV140_HUMAN	4,745004	7,476273E-09
P01709	Immunoglobulin lambda variable 2-8	LV208_HUMAN	4,507547	6,532884E-06
P01714	Immunoglobulin lambda variable 3-19	LV319_HUMAN	4,378443	7,476273E-09
P01717	Immunoglobulin lambda variable 3-25	LV325_HUMAN	6,128181	1,648543E-08
P01742	Immunoglobulin heavy variable 1-69	HV169_HUMAN	5,937338	2,420401E-08
P01766	Immunoglobulin heavy variable 3-13	HV313_HUMAN	3,180457	4,220997E-06
P01768	Immunoglobulin heavy variable 3-30	HV330_HUMAN	6,823731	1,452633E-08
P0DP03	Immunoglobulin heavy variable 3-30-5	HVC05_HUMAN	6,823731	1,452633E-08
P01780	Immunoglobulin heavy variable 3-7	HV307_HUMAN	8,795857	1,908095E-07

P01782	Immunoglobulin heavy variable 3-9	HV309_HUMAN	5,586367	6,247050E-08
P0DP04	Immunoglobulin heavy variable 3-43D	HV43D_HUMAN	5,586367	6,247050E-08
P01817	Immunoglobulin heavy variable 2-5	HV205_HUMAN	3,285530	3,299972E-07
P01833	Polymeric immunoglobulin receptor	PIGR_HUMAN	14,937788	7,476273E-09
P01834	Immunoglobulin kappa constant	IGKC_HUMAN	12,739011	7,318284E-08
P01859	Immunoglobulin heavy constant gamma 2	IGHG2_HUMAN	5,839009	1,928103E-07
P01871	Immunoglobulin heavy constant mu	IGHM_HUMAN	10,267075	7,476273E-09
P01876	Immunoglobulin heavy constant alpha 1	IGHA1_HUMAN	15,537470	7,476273E-09
P01877	Immunoglobulin heavy constant alpha 2	IGHA2_HUMAN	15,764662	1,086402E-07
P02533	Keratin, type I cytoskeletal 14	K1C14_HUMAN	3,487919	1,311250E-08
P02538	Keratin, type II cytoskeletal 6A	K2C6A_HUMAN	4,372670	7,476273E-09
P02647	Apolipoprotein A-I	APOA1_HUMAN	4,671760	7,476273E-09
P02649	Apolipoprotein E	APOE_HUMAN	2,525491	3,480639E-07
P02656	Apolipoprotein C-III	APOC3_HUMAN	5,206449	4,227246E-05
P02671	Fibrinogen alpha chain	FIBA_HUMAN	2,228236	6,700566E-07
P02675	Fibrinogen beta chain	FIBB_HUMAN	3,968231	4,838005E-08
P02679	Fibrinogen gamma chain	FIBG_HUMAN	3,752473	1,589505E-07
P02749	Beta-2-glycoprotein 1	APOH_HUMAN	3,422151	3,834623E-08
P02750	Leucine-rich alpha-2-glycoprotein	A2GL_HUMAN	5,056493	7,476273E-09
P02763	Alpha-1-acid glycoprotein 1	A1AG1_HUMAN	4,198172	7,476273E-09
P02765	Alpha-2-HS-glycoprotein	FETUA_HUMAN	3,323301	2,995563E-06
P02766	Transthyretin	TTHY_HUMAN	3,556298	7,476273E-09
P02768	Albumin	ALBU_HUMAN	11,840419	7,476273E-09
P02774	Vitamin D-binding protein	VTDB_HUMAN	4,122363	6,895466E-08
P02787	Serotransferrin	TRFE_HUMAN	5,780000	7,476273E-09
P02788	Lactotransferrin	TRFL_HUMAN	17,394282	7,476273E-09
P02790	Hemopexin	HEMO_HUMAN	5,037239	2,068216E-07
P03950	Angiogenin	ANGI_HUMAN	2,354979	8,753205E-07

P03973	Antileukoproteinase	SLPI_HUMAN	7,791596	7,476273E-09
P04004	Vitronectin	VTNC_HUMAN	2,696379	3,211609E-08
P04066	Tissue alpha-L-fucosidase	FUCO_HUMAN	3,061654	7,476273E-09
P04075	Fructose-bisphosphate aldolase A	ALDOA_HUMAN	2,164514	8,379444E-09
P04080	Cystatin-B	CYTB_HUMAN	2,213534	6,794157E-05
P04083	Annexin A1	ANXA1_HUMAN	3,480413	8,467789E-09
P04211	Immunoglobulin lambda variable 7-43	LV743_HUMAN	8,141720	7,476273E-09
P04217	Alpha-1B-glycoprotein	A1BG_HUMAN	5,353072	2,915400E-04
P04264	Keratin, type II cytoskeletal 1	K2C1_HUMAN	7,479547	7,476273E-09
P04406	Glyceraldehyde-3-phosphate dehydrogenase	G3P_HUMAN	5,198018	4,230109E-07
P04792	Heat shock protein beta-1	HSPB1_HUMAN	5,206262	7,476273E-09
P04908	Histone H2A type 1-B/E	H2A1B_HUMAN	5,229340	7,476273E-09
P0C0S8	Histone H2A type 1	H2A1_HUMAN	5,229340	7,476273E-09
P16104	Histone H2AX	H2AX_HUMAN	5,229340	7,476273E-09
P20671	Histone H2A type 1-D	H2A1D_HUMAN	5,229340	7,476273E-09
Q16777	Histone H2A type 2-C	H2A2C_HUMAN	5,229340	7,476273E-09
Q6FI13	Histone H2A type 2-A	H2A2A_HUMAN	5,229340	7,476273E-09
Q7L7L0	Histone H2A type 3	H2A3_HUMAN	5,229340	7,476273E-09
Q93077	Histone H2A type 1-C	H2A1C_HUMAN	5,229340	7,476273E-09
Q96KK5	Histone H2A type 1-H	H2A1H_HUMAN	5,229340	7,476273E-09
Q96QV6	Histone H2A type 1-A	H2A1A_HUMAN	5,229340	7,476273E-09
Q99878	Histone H2A type 1-J	H2A1J_HUMAN	5,229340	7,476273E-09
Q9BTM1	Histone H2A.J	H2AJ_HUMAN	5,229340	7,476273E-09
P05089	Arginase-1	ARGI1_HUMAN	9,059025	3,222278E-07
P05090	Apolipoprotein D	APOD_HUMAN	5,759071	2,198924E-07
P05109	Protein S100-A8	S10A8_HUMAN	7,012274	1,141530E-08
P05155	Plasma protease C1 inhibitor	IC1_HUMAN	4,039042	3,528370E-08
P05164	Myeloperoxidase	PERM_HUMAN	3,311214	8,787303E-08

P05783	Keratin, type I cytoskeletal 18	K1C18_HUMAN	6,923160	3,536393E-07
P05814	Beta-casein	CASB_HUMAN	6,260000	4,378668E-07
P06312	Immunoglobulin kappa variable 4-1	KV401_HUMAN	8,117072	3,646868E-08
P06396	Gelsolin	GELS_HUMAN	7,199160	7,476273E-09
P06702	Protein S100-A9	S10A9_HUMAN	6,038862	6,666783E-08
P06703	Protein S100-A6	S10A6_HUMAN	5,928129	1,793575E-07
P06727	Apolipoprotein A-IV	APOA4_HUMAN	2,698879	1,755999E-07
P06733	Alpha-enolase	ENOA_HUMAN	8,246886	1,694282E-07
P06865	Beta-hexosaminidase subunit alpha	HEXA_HUMAN	2,370653	3,565187E-06
P07237	Protein disulfide-isomerase	PDIA1_HUMAN	2,079225	1,359249E-06
P07339	Cathepsin D	CATD_HUMAN	6,410000	7,476273E-09
P07355	Annexin A2	ANXA2_HUMAN	3,485210	5,218377E-08
P07437	Tubulin beta chain	TBB5_HUMAN	2,154783	3,199707E-07
P07477	Trypsin-1	TRY1_HUMAN	4,653675	1,624214E-06
Q9BYE2	Transmembrane protease serine 13	TMPSD_HUMAN	4,653675	1,624214E-06
P07602	Prosaposin	SAP_HUMAN	6,187491	1,007703E-08
P07686	Beta-hexosaminidase subunit beta	HEXB_HUMAN	2,684376	1,993916E-08
P07711	Procathepsin L	CATL1_HUMAN	5,384087	2,440872E-05
P07737	Profilin-1	PROF1_HUMAN	3,661594	2,169109E-07
P07858	Cathepsin B	CATB_HUMAN	5,740994	1,737387E-07
P08174	Complement decay-accelerating factor	DAF_HUMAN	1,091352	5,225243E-06
P08246	Neutrophil elastase	ELNE_HUMAN	4,008797	7,391850E-07
P08294	Extracellular superoxide dismutase [Cu-Zn]	SODE_HUMAN	1,402113	4,093388E-06
P08311	Cathepsin G	CATG_HUMAN	2,890000	7,476273E-09
P08571	Monocyte differentiation antigen CD14	CD14_HUMAN	4,516079	7,476273E-09
P08582	Melanotransferrin	TRFM_HUMAN	1,768809	7,476273E-09
P08727	Keratin, type I cytoskeletal 19	K1C19_HUMAN	7,543563	7,476273E-09
P08758	Annexin A5	ANXA5_HUMAN	4,226451	7,476273E-09

P09211	Glutathione S-transferase P	GSTP1_HUMAN	5,098389	2,631949E-07
P09228	Cystatin-SA	CYTT_HUMAN	6,858357	3,633000E-07
P0C0L4	Complement C4-A	CO4A_HUMAN	1,991840	7,476273E-09
P0CG47	Polyubiquitin-B	UBB_HUMAN	4,516537	1,362744E-05
P0CG48	Polyubiquitin-C	UBC_HUMAN	4,516537	1,362744E-05
P62979	Ubiquitin-40S ribosomal protein S27a	RS27A_HUMAN	4,516537	1,362744E-05
P62987	Ubiquitin-60S ribosomal protein L40	RL40_HUMAN	4,516537	1,362744E-05
P0DMV8	Heat shock 70 kDa protein 1A	HS71A_HUMAN	3,816331	1,045384E-08
P0DMV9	Heat shock 70 kDa protein 1B	HS71B_HUMAN	3,816331	1,045384E-08
P0DOX2	Immunoglobulin alpha-2 heavy chain	IGA2_HUMAN	13,998466	8,041104E-09
P0DOX3	Immunoglobulin delta heavy chain	IGD_HUMAN	6,160000	7,476273E-09
P0DOX4	Immunoglobulin epsilon heavy chain	IGE_HUMAN	5,461588	7,341474E-06
P0DOX5	Immunoglobulin gamma-1 heavy chain	IGG1_HUMAN	7,720000	7,476273E-09
P0DOX7	Immunoglobulin kappa light chain	IGK_HUMAN	11,164677	7,476273E-09
P0DOX8	Immunoglobulin lambda-1 light chain	IGL1_HUMAN	10,981307	2,601259E-08
P0DOY2	Immunoglobulin lambda constant 2	IGLC2_HUMAN	12,787421	6,139125E-07
P0DOY3	Immunoglobulin lambda constant 3	IGLC3_HUMAN	12,787421	6,139125E-07
P0DTE7	Alpha-amylase 1B	AMY1B_HUMAN	2,558030	7,476273E-09
P0DTE8	Alpha-amylase 1C	AMY1C_HUMAN	2,558030	7,476273E-09
P0DUB6	Alpha-amylase 1A	AMY1A_HUMAN	2,558030	7,476273E-09
P10412	Histone H1.4	H14_HUMAN	2,114343	7,892122E-08
P16402	Histone H1.3	H13_HUMAN	2,114343	7,892122E-08
P16403	Histone H1.2	H12_HUMAN	2,114343	7,892122E-08
P10599	Thioredoxin	THIO_HUMAN	3,677969	9,859280E-08
P10909	Clusterin	CLUS_HUMAN	9,447152	7,476273E-09
P11021	Endoplasmic reticulum chaperone BiP	BIP_HUMAN	2,662800	1,096698E-07
P11142	Heat shock cognate 71 kDa protein	HSP7C_HUMAN	1,799871	9,025233E-07
P12273	Prolactin-inducible protein	PIP_HUMAN	14,386432	1,749203E-08

P12429	Annexin A3	ANXA3_HUMAN	4,966176	2,024093E-07
P12830	Cadherin-1	CADH1_HUMAN	5,142440	7,895579E-08
P13639	Elongation factor 2	EF2_HUMAN	1,758054	1,273206E-06
P13645	Keratin, type I cytoskeletal 10	K1C10_HUMAN	6,361607	7,476273E-09
P13646	Keratin, type I cytoskeletal 13	K1C13_HUMAN	7,953648	7,476273E-09
P13647	Keratin, type II cytoskeletal 5	K2C5_HUMAN	5,276788	7,476273E-09
P13688	Carcinoembryonic antigen-related cell adhesion molecule 1	CEAM1_HUMAN	4,294853	4,598480E-04
P13796	Plastin-2	PLSL_HUMAN	3,676020	3,056804E-07
P13987	CD59 glycoprotein	CD59_HUMAN	4,102317	7,419733E-07
P14174	Macrophage migration inhibitory factor	MIF_HUMAN	3,015013	1,550290E-05
P14314	Glucosidase 2 subunit beta	GLU2B_HUMAN	2,768065	5,795173E-07
P14555	Phospholipase A2, membrane associated	PA2GA_HUMAN	9,710000	7,476273E-09
P14618	Pyruvate kinase PKM	KPYM_HUMAN	4,530036	7,476273E-09
P15311	Ezrin	EZRI_HUMAN	4,351799	7,476273E-09
P15531	Nucleoside diphosphate kinase A	NDKA_HUMAN	2,331288	4,769184E-06
P22392	Nucleoside diphosphate kinase B	NDKB_HUMAN	2,331288	4,769184E-06
P16144	Integrin beta-4	ITB4_HUMAN	6,683021	1,373296E-03
P16520	Guanine nucleotide-binding protein G(I)/G(S)/G(T) subunit beta-3	GBB3_HUMAN	2,695633	2,986873E-07
P62873	Guanine nucleotide-binding protein G(I)/G(S)/G(T) subunit beta-1	GBB1_HUMAN	2,695633	2,986873E-07
P62879	Guanine nucleotide-binding protein G(I)/G(S)/G(T) subunit beta-2	GBB2_HUMAN	2,695633	2,986873E-07
Q9HAV0	Guanine nucleotide-binding protein subunit beta-4	GBB4_HUMAN	2,695633	2,986873E-07
P17900	Ganglioside GM2 activator	SAP3_HUMAN	2,175401	7,355164E-07
P17931	Galectin-3	LEG3_HUMAN	4,370000	7,476273E-09
P19013	Keratin, type II cytoskeletal 4	K2C4_HUMAN	6,000630	2,137204E-08
P19021	Peptidyl-glycine alpha-amidating monooxygenase	AMD_HUMAN	1,750094	4,595863E-06
P19652	Alpha-1-acid glycoprotein 2	A1AG2_HUMAN	4,234991	2,461935E-06
P19971	Thymidine phosphorylase	TYPH_HUMAN	7,923549	6,148481E-07
P20061	Transcobalamin-1	TCO1_HUMAN	9,841223	8,216933E-08

P20160	Azurocidin	CAP7_HUMAN	3,986598	1,691095E-06
P21926	CD9 antigen	CD9_HUMAN	1,949765	1,975287E-08
P21980	Protein-glutamine gamma-glutamyltransferase 2	TGM2_HUMAN	2,638238	1,173666E-06
P22079	Lactoperoxidase	PERL_HUMAN	7,798307	7,476273E-09
P22352	Glutathione peroxidase 3	GPX3_HUMAN	3,920441	4,210842E-06
P23083	Immunoglobulin heavy variable 1-2	HV102_HUMAN	7,524494	1,126309E-07
P23284	Peptidyl-prolyl cis-trans isomerase B	PPIB_HUMAN	4,176942	7,476273E-09
P23443	Ribosomal protein S6 kinase beta-1	KS6B1_HUMAN	3,149412	1,717860E-04
P23528	Cofilin-1	COF1_HUMAN	3,490956	1,181078E-05
P24158	Myeloblastin	PRTN3_HUMAN	2,917573	1,234024E-08
P25311	Zinc-alpha-2-glycoprotein	ZA2G_HUMAN	13,990314	7,476273E-09
P25774	Cathepsin S	CATS_HUMAN	2,466620	1,439141E-07
P25815	Protein S100-P	S100P_HUMAN	3,605958	9,921694E-08
P26447	Protein S100-A4	S10A4_HUMAN	2,848693	7,476273E-09
P27105	Stomatin	STOM_HUMAN	3,278284	1,683528E-07
P27487	Dipeptidyl peptidase 4	DPP4_HUMAN	2,500000	2,254024E-06
P28799	Progranulin	GRN_HUMAN	3,974436	4,104862E-07
P29401	Transketolase	TKT_HUMAN	2,250618	6,593357E-07
P30041	Peroxiredoxin-6	PRDX6_HUMAN	3,127178	3,434829E-07
P30044	Peroxiredoxin-5, mitochondrial	PRDX5_HUMAN	3,439458	5,516446E-07
P30101	Protein disulfide-isomerase A3	PDIA3_HUMAN	5,498260	8,966821E-05
P30740	Leukocyte elastase inhibitor	ILEU_HUMAN	3,437653	7,476273E-09
P30838	Aldehyde dehydrogenase, dimeric NADP-preferring	AL3A1_HUMAN	2,189088	3,237388E-06
P31025	Lipocalin-1	LCN1_HUMAN	17,974692	7,476273E-09
P31949	Protein S100-A11	S10AB_HUMAN	2,793423	7,476273E-09
P34096	Ribonuclease 4	RNAS4_HUMAN	3,396448	7,476273E-09
P35527	Keratin, type I cytoskeletal 9	K1C9_HUMAN	5,265886	7,476273E-09
P35749	Myosin-11	MYH11_HUMAN	7,277051	5,021044E-05

P35908	Keratin, type II cytoskeletal 2 epidermal	K22E_HUMAN	5,342344	7,476273E-09
P36955	Pigment epithelium-derived factor	PEDF_HUMAN	2,790403	1,754664E-08
P37802	Transgelin-2	TAGL2_HUMAN	1,684900	3,525975E-06
P40199	Carcinoembryonic antigen-related cell adhesion molecule 6	CEAM6_HUMAN	2,403927	7,476273E-09
P42330	Aldo-keto reductase family 1 member C3	AK1C3_HUMAN	2,399427	2,021120E-06
Q04828	Aldo-keto reductase family 1 member C1	AK1C1_HUMAN	2,399427	2,021120E-06
P43251	Biotinidase	BTD_HUMAN	8,137857	4,589302E-06
P45877	Peptidyl-prolyl cis-trans isomerase C	PPIC_HUMAN	5,515309	5,420948E-05
P48668	Keratin, type II cytoskeletal 6C	K2C6C_HUMAN	7,166400	7,476273E-09
P48723	Heat shock 70 kDa protein 13	HSP13_HUMAN	2,969564	2,932414E-08
P49327	Fatty acid synthase	FAS_HUMAN	3,257261	2,080854E-03
P49788	Retinoic acid receptor responder protein 1	TIG1_HUMAN	5,739197	7,476273E-09
P52209	6-phosphogluconate dehydrogenase, decarboxylating	6PGD_HUMAN	3,029620	7,476273E-09
P52566	Rho GDP-dissociation inhibitor 2	GDIR2_HUMAN	3,210237	3,047038E-06
P53634	Dipeptidyl peptidase 1	CATC_HUMAN	3,726084	1,014431E-08
P54252	Ataxin-3	ATX3_HUMAN	4,158252	8,594153E-06
P55058	Phospholipid transfer protein	PLTP_HUMAN	6,972728	7,476273E-09
P55064	Aquaporin-5	AQP5_HUMAN	3,628811	9,221656E-06
P58107	Epiplakin	EPIPL_HUMAN	4,634313	4,834839E-06
P58499	Protein FAM3B	FAM3B_HUMAN	2,340699	2,773465E-05
P59665	Neutrophil defensin 1	DEF1_HUMAN	4,814479	2,695581E-08
P59666	Neutrophil defensin 3	DEF3_HUMAN	4,814479	2,695581E-08
P59998	Actin-related protein 2/3 complex subunit 4	ARPC4_HUMAN	2,061387	1,775861E-06
P60174	Triosephosphate isomerase	TPIS_HUMAN	2,960000	7,476273E-09
P60709	Actin, cytoplasmic 1	ACTB_HUMAN	7,510687	1,270795E-08
P63261	Actin, cytoplasmic 2	ACTG_HUMAN	7,510687	1,270795E-08
P61224	Ras-related protein Rap-1b	RAP1B_HUMAN	2,365354	2,779948E-06
P62834	Ras-related protein Rap-1A	RAP1A_HUMAN	2,365354	2,779948E-06

P61626	Lysozyme C	LYSC_HUMAN	17,203636	8,838014E-09
P61769	Beta-2-microglobulin	B2MG_HUMAN	7,812270	1,516169E-08
P62258	14-3-3 protein epsilon	1433E_HUMAN	4,968489	1,687958E-08
P62805	Histone H4	H4_HUMAN	5,166633	7,893949E-08
P62937	Peptidyl-prolyl cis-trans isomerase A	PPIA_HUMAN	2,998800	1,239815E-08
P63104	14-3-3 protein zeta/delta	1433Z_HUMAN	3,174170	2,789985E-06
P68104	Elongation factor 1-alpha 1	EF1A1_HUMAN	4,384235	2,073643E-07
Q5VTE0	Putative elongation factor 1-alpha-like 3	EF1A3_HUMAN	4,384235	2,073643E-07
P68363	Tubulin alpha-1B chain	TBA1B_HUMAN	2,568856	2,271324E-07
Q71U36	Tubulin alpha-1A chain	TBA1A_HUMAN	2,568856	2,271324E-07
Q9BQE3	Tubulin alpha-1C chain	TBA1C_HUMAN	2,568856	2,271324E-07
P68871	Hemoglobin subunit beta	HBB_HUMAN	3,424078	9,306335E-09
P69905	Hemoglobin subunit alpha	HBA_HUMAN	2,152217	7,301521E-07
P80188	Neutrophil gelatinase-associated lipocalin	NGAL_HUMAN	7,257606	7,476273E-09
P80303	Nucleobindin-2	NUCB2_HUMAN	7,194292	2,479433E-08
P81605	Dermcidin	DCD_HUMAN	2,337526	5,447596E-07
P98160	Basement membrane-specific heparan sulfate proteoglycan core protein	PGBM_HUMAN	3,919425	7,476273E-09
Q01469	Fatty acid-binding protein 5	FABP5_HUMAN	3,560685	1,998752E-07
Q02487	Desmocollin-2	DSC2_HUMAN	1,992864	1,072948E-06
Q02809	Procollagen-lysine,2-oxoglutarate 5-dioxygenase 1	PLOD1_HUMAN	3,100452	7,476273E-09
Q02818	Nucleobindin-1	NUCB1_HUMAN	4,210000	8,558008E-09
Q04609	Glutamate carboxypeptidase 2	FOLH1_HUMAN	2,527845	1,275290E-04
Q9HBA9	Putative N-acetylated-alpha-linked acidic dipeptidase	FOH1B_HUMAN	2,527845	1,275290E-04
Q04917	14-3-3 protein eta	1433F_HUMAN	3,305000	1,747580E-04
Q06830	Peroxiredoxin-1	PRDX1_HUMAN	4,788790	7,476273E-09
Q08380	Galectin-3-binding protein	LG3BP_HUMAN	8,507704	7,476273E-09
Q08431	Lactadherin	MFGM_HUMAN	3,850589	7,476273E-09
Q13162	Peroxiredoxin-4	PRDX4_HUMAN	2,523732	9,082490E-04

Q13217	DnaJ homolog subfamily C member 3	DNJC3_HUMAN	5,410603	1,762889E-08
Q13228	Methanethiol oxidase	SBP1_HUMAN	3,321895	3,164413E-08
Q13296	Mammaglobin-A	SG2A2_HUMAN	6,917646	2,881435E-06
Q13421	Mesothelin	MSLN_HUMAN	8,822283	7,476273E-09
Q13510	Acid ceramidase	ASAH1_HUMAN	1,837557	4,454384E-08
Q13867	Bleomycin hydrolase	BLMH_HUMAN	2,795301	7,624680E-07
Q14002	Carcinoembryonic antigen-related cell adhesion molecule 7	CEAM7_HUMAN	2,139104	2,378640E-07
Q14116	Interleukin-18	IL18_HUMAN	3,891729	1,533261E-05
Q14118	Dystroglycan 1	DAG1_HUMAN	4,101051	1,306040E-06
Q14515	SPARC-like protein 1	SPRL1_HUMAN	4,476353	1,834854E-06
Q14697	Neutral alpha-glucosidase AB	GANAB_HUMAN	4,385107	7,476273E-09
Q15293	Reticulocalbin-1	RCN1_HUMAN	1,899655	7,476273E-09
Q15782	Chitinase-3-like protein 2	CH3L2_HUMAN	5,230191	5,060847E-07
Q16378	Proline-rich protein 4	PROL4_HUMAN	12,629968	2,157168E-08
Q16651	Prostasin	PRSS8_HUMAN	5,076379	3,932702E-07
Q687X5	Metalloreductase STEAP4	STEA4_HUMAN	1,788849	6,708576E-06
Q6MZM9	Proline-rich protein 27	PRR27_HUMAN	6,349401	1,256011E-07
Q6WRX3	Protein zyg-11 homolog A	ZY11A_HUMAN	6,716317	5,073290E-04
Q6ZRV2	Protein FAM83H	FA83H_HUMAN	4,427418	1,405920E-04
Q86X29	Lipolysis-stimulated lipoprotein receptor	LSR_HUMAN	2,743338	7,987953E-08
Q8IVU3	Probable E3 ubiquitin-protein ligase HERC6	HERC6_HUMAN	3,465355	5,487776E-05
Q8IZ81	ELMO domain-containing protein 2	ELMD2_HUMAN	8,462970	5,626341E-07
Q8N474	Secreted frizzled-related protein 1	SFRP1_HUMAN	4,926503	6,235021E-08
Q8N7B9	EF-hand calcium-binding domain-containing protein 3	EFCB3_HUMAN	5,205305	6,740690E-04
Q8N8E3	Centrosomal protein of 112 kDa	CE112_HUMAN	2,978268	1,405870E-04
Q8N8K9	Uncharacterized protein KIAA1958	K1958_HUMAN	4,519886	2,078318E-06
Q8NBJ4	Golgi membrane protein 1	GOLM1_HUMAN	3,333024	3,292100E-08
Q8NCL4	Polypeptide N-acetylgalactosaminyltransferase 6	GALT6_HUMAN	2,600435	9,538309E-07

Q8NEM7	Transcription factor SPT20 homolog	SP20H_HUMAN	9,482262	1,791147E-03
Q8NES3	Beta-1,3-N-acetylglucosaminyltransferase lunatic fringe	LFNG_HUMAN	1,756531	3,161860E-04
Q8NFU4	Follicular dendritic cell secreted peptide	FDSCP_HUMAN	3,447478	9,564201E-06
Q8TCU4	Alstrom syndrome protein 1	ALMS1_HUMAN	5,864483	1,023834E-03
Q8TEQ8	GPI ethanolamine phosphate transferase 3	PIGO_HUMAN	12,712316	3,277360E-04
Q8WUM4	Programmed cell death 6-interacting protein	PDC6I_HUMAN	1,311025	6,999840E-04
Q8WVN6	Secreted and transmembrane protein 1	SCTM1_HUMAN	5,120000	6,304180E-04
Q8WVQ1	Soluble calcium-activated nucleotidase 1	CANT1_HUMAN	1,988717	2,282270E-04
Q8WXG9	Adhesion G-protein coupled receptor V1	AGRV1_HUMAN	3,185004	3,388241E-07
Q8WZ42	Titin	TITIN_HUMAN	6,334398	1,759874E-05
Q8WZA2	Rap guanine nucleotide exchange factor 4	RPGF4_HUMAN	6,211699	2,088150E-04
Q92743	Serine protease HTRA1	HTRA1_HUMAN	5,175037	7,476273E-09
Q92784	Zinc finger protein DPF3	DPF3_HUMAN	6,753299	1,718264E-06
Q92932	Receptor-type tyrosine-protein phosphatase N2	PTPR2_HUMAN	9,663069	4,946630E-04
Q96BQ1	Protein FAM3D	FAM3D_HUMAN	1,738102	1,478560E-04
Q96DA0	Zymogen granule protein 16 homolog B	ZG16B_HUMAN	11,170132	1,166234E-08
Q96DR8	Mucin-like protein 1	MUCL1_HUMAN	3,057122	2,167599E-07
Q96S96	Phosphatidylethanolamine-binding protein 4	PEBP4_HUMAN	2,646231	1,180141E-05
Q96ST3	Paired amphipathic helix protein Sin3a	SIN3A_HUMAN	7,434919	7,197220E-04
Q99519	Sialidase-1	NEUR1_HUMAN	2,779718	5,784983E-07
Q99574	Neuroserpin	NEUS_HUMAN	4,045589	5,034094E-07
Q99935	Opiorphin prepropeptide	PROL1_HUMAN	12,178373	6,430223E-08
Q99954	Submaxillary gland androgen-regulated protein 3A	SMR3A_HUMAN	6,235035	3,670500E-05
Q9BRK5	45 kDa calcium-binding protein	CAB45_HUMAN	2,858488	7,338341E-07
Q9GZN4	Brain-specific serine protease 4	BSSP4_HUMAN	2,667296	5,375260E-05
Q9GZZ8	Extracellular glycoprotein lacritin	LACRT_HUMAN	15,058896	7,476273E-09
Q9H792	Inactive tyrosine-protein kinase PEAK1	PEAK1_HUMAN	5,374786	1,032870E-04
Q9H9G7	Protein argonaute-3	AGO3_HUMAN	3,490469	1,188906E-06

Q9H9H5	MAP6 domain-containing protein 1	MA6D1_HUMAN	2,467342	2,134517E-05
Q9NPD7	Neuritin	NRN1_HUMAN	4,800640	8,294413E-05
Q9NRA1	Platelet-derived growth factor C	PDGFC_HUMAN	2,926416	8,920643E-07
Q9UBR2	Cathepsin Z	CATZ_HUMAN	2,095738	1,132730E-04
Q9UGB7	Inositol oxygenase	MIOX_HUMAN	2,197016	1,272355E-03
Q9UGF5	Olfactory receptor 14J1	O14J1_HUMAN	3,277640	1,415820E-04
Q9UGM3	Deleted in malignant brain tumors 1 protein	DMBT1_HUMAN	6,741754	7,476273E-09
Q9UJJ9	N-acetylglucosamine-1-phosphotransferase subunit gamma	GNPTG_HUMAN	2,416366	1,794526E-03
Q9UJV9	Probable ATP-dependent RNA helicase DDX41	DDX41_HUMAN	3,548643	3,085941E-05
Q9Y4L1	Hypoxia up-regulated protein 1	HYOU1_HUMAN	2,046151	5,624180E-05
Q9Y6N1	Cytochrome c oxidase assembly protein COX11, mitochondrial	COX11_HUMAN	3,220000	4,171584E-05
Q9Y6N6	Laminin subunit gamma-3	LAMC3_HUMAN	5,814233	6,877585E-05
